# Acute pancreatitis in immunocompromised patients: beware of varicella zoster virus primo‐infection

**DOI:** 10.1002/ccr3.1053

**Published:** 2017-06-22

**Authors:** Adrien Picod, Elise Corre, Eric Maury, Paul Duriez, Nadia Hoyeau, Paul Coppo

**Affiliations:** ^1^ Service d'Hématologie Groupe Hospitalier Paris‐Est Hôpital Saint‐Antoine Paris France; ^2^ Service de Réanimation polyvalente Groupe Hospitalier Paris‐Est Hôpital Saint‐Antoine Paris France; ^3^ Université Pierre et Marie Curie (UPMC) Univ Paris 6 Paris France; ^4^ Service d'Anatomie et cytologie pathologique Groupe Hospitalier Paris‐Est Hôpital Saint‐Antoine Paris France; ^5^ Centre de références des Microangiopathies thrombotiques Groupe Hospitalier Paris‐Est Hôpital Saint‐Antoine Paris France; ^6^ Inserm U1170 Institut Gustave Roussy Villejuif France

**Keywords:** Chronic lymphocytic leukemia, human herpesvirus 3, inappropriate ADH syndrome, pancreatitis

## Abstract

Varicella zoster virus (VZV) primo‐infection can be severe in the elderly and in immunocompromised patients. Atypical presentations are not uncommon and may mislead the diagnosis and delay adequate treatment. Valacyclovir prophylaxis should be systematically proposed in immunocompromised patients.

## Introduction

Varicella zoster virus (VZV) primo‐infection in immunocompromised patients is a rare and potentially serious event. Prognosis is dismal even with adequate treatment. We report an atypical case of fatal varicella primo‐infection in an immunocompromised elderly female. Clinicians should be aware of this diagnosis and its atypical presentations for a rapid adapted management.

## Case History

A 86‐year‐old female with a history of chronic lymphocytic leukemia presented with diffuse abdominal pain, nausea, and vomiting. She had been treated by an immunochemotherapy regimen associating rituximab, fludarabine, and cyclophosphamide 3 years ago with a very good partial remission and was as without specific treatment. She had no daily medication.

Upon admission, her clinical examination was unremarkable. She had no fever and denied any recent contact with an ill person. Except for lymphocytosis related to the underlying malignancy, initial laboratory screening was remarkable for hyponatremia (123 mmol/L) with low plasma osmolality (272 mOsm/kg H2O) and inappropriately high urine osmolality (863 mOsm/kg H2O). Serum creatinine was normal (73 *μ*mol/L), and her sodium excretion was elevated. These findings were consistent with inappropriate antidiuretic hormone secretion.

Initial evaluation in the emergency department excluded surgical emergency, and she was admitted for further investigations. Serum lipase slowly rose above 3‐time the normal value, up to 1131 UI/L (normal range 0–67 UI/L) leading to the diagnosis of acute pancreatitis. Due to persistent pain, oral nutrition was suspended.

**Figure 1:**
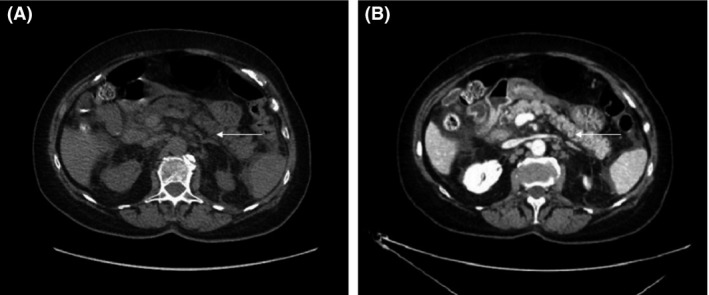
Computed tomography without (A) and after injection, early portal phase (B). Slight enlargement of pancreas (white arrows) and moderate infiltration of peripancreatic fat.

The computed tomography (CT) performed 48 h after diagnosis showed mild pancreatitis with enlargement of pancreas and moderate infiltration of peripancreatic fat (Balthazar score C, CT severity index 4). There was neither pancreatic necrosis nor peripancreatic collection (Fig. 1).

She denied any alcohol consumption. Trans‐abdominal ultrasound examination was normal. Magnetic resonance cholangiopancreatography was performed and showed no biliary obstruction. Serum calcium and lipid were within normal range, and investigations were directed toward a rare cause of pancreatitis (i.e., immune or infectious).

**Figure 2:**
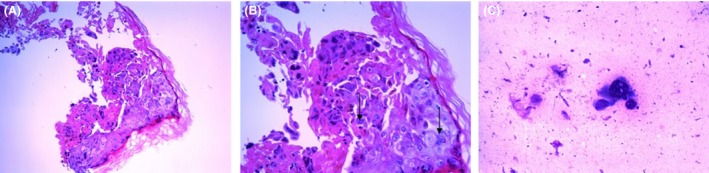
Cytopathogenic effect on skin biopsy, hematoxylin‐eosin staining: epidermic necrosis (white arrow), multinucleated cells (black arrows) (A) and (B); Tzanck smear, May‐Grünwald Giemsa staining: dystrophic nuclei, ground glass chromatin (C).

Her condition progressively deteriorated, with worsening pain, impaired mental status, drowsiness, and confusion. Five days after her admission she suddenly demonstrated acute respiratory distress and hypotension with mottling. She was admitted in the intensive care unit, placed under mechanical ventilation, and required a vasopressor support. Repeated CT was unremarkable except for low abundance ascites. Rare vesicles on cutaneous examination suggested a herpes virus infection. Tzanck smear and skin biopsy were performed, and blood, ascites, broncho‐alveolar fluid, and cerebrospinal fluid were sampled for herpesvirus. Despite empiric intravenous acyclovir therapy, her condition deteriorated and she died in a context of multiorgan failure 48 h after her admission in the intensive care unit. The identification of viral DNA in blood, broncho‐alveolar fluid, ascites, and cerebrospinal fluid confirmed the disseminated infection by VZV. No other pathogen was found in these samples. Tzanck smear and skin biopsy showed cytopathogenic effect (Fig. 2). A negative VZV serology was consistent with a primo‐infection.

## Discussion

Varicella zoster virus or human herpes virus 3 is an alpha‐herpes virus responsible for varicella or chickenpox and herpes zoster. Most primo‐infections occur in early childhood, leading to a usually benign, self‐limited illness in the immunocompetent people. Symptoms include moderate fever, malaise, and typical skin rash with maculopapules and vesicles in varying stages of evolution. In France, the seroprevalence by adult life is >90% 1. Although rare, primo‐infection in adults may be severe, with a greater occurrence of visceral complication such as pneumonia. Infants, adults, pregnant women, and immunocompromised patients are particularly at risk of progressive, life‐threatening disease with multiorgan involvement 2. Among the elderly, data are scarce given the rarity of this situation, but it can be assumed that the greater frequency of comorbidity is a pejorative risk factor.

Our case highlights the extreme gravity of a VZV primo‐infection in immunocompromised elderly patients. Several learning points should be emphasized. First, the absence of fever does not exclude the diagnosis. Indeed, our patient remained without fever for the whole clinical course. Furthermore, the infection can be revealed by an unusual manifestation 3, 4, such as acute pancreatitis, myocarditis, pneumonia, or encephalitis. Second, the apparent absence of contact with a case of varicella does not systematically exclude the diagnosis either. Finally, the characteristic skin vesicles may be absent or delayed. If skin lesions are present, Tzanck smear may be useful by demonstrating a cytopathogenic effect. Acute abdominal pain as initial presentation of varicella or disseminated herpes zoster has already been described sometimes associated with inappropriate antidiuretic hormone secretion 5. Direct infection of the muscularis propria and myenteric plexi by VZV, as previously demonstrated 6 might explain in our case the onset of abdominal pain several days before serum lipase elevation.

Early treatment can reverse the unfavorable clinical course, but as the diagnosis may be challenging, efforts must be made to prevent the infection among high‐risk patients. VZV immunization should be sought by questioning and serology. The live attenuated vaccine is conventionally contraindicated in immunocompromised patients. Although some patients with humoral immunosuppression have been successfully and uncomplicatedly vaccinated, there is currently no recommendation on the vaccination of adult patients suffering from hematologic malignancies. As most unimmunized immunocompromised patients cannot receive the live attenuated vaccine, valacyclovir prophylaxis should be proposed systematically in this population 7.

## Conclusion

Varicella zoster virus primo‐infection in immunocompromised patients can lead to a rapidly progressive, life‐threatening disease. Clinicians should be aware of this diagnosis in immunocompromised patients with atypical presentations such as acute pancreatitis, which may still worsen prognosis by delaying diagnosis and treatment initiation. Unimmunized patients ineligible for vaccination such as patients with hematologic malignancies should receive oral valacyclovir prophylaxis and benefit from appropriate care in case of exposure.

## Authorship

AP: collected data and drafted the manuscript. PD and NH: provided images and pathology description. EC, EM, and PC: collected data and corrected the manuscript. All the authors approved the final version.

## Conflict of Interest

None declared.

## Consent

Informed consent for the report was obtained from the patient's family.
